# Adult Refsum Disease: Case Series of Reducing Circulating Phytanic Acid Levels With Dietary Interventions

**DOI:** 10.1002/jmd2.70048

**Published:** 2026-05-15

**Authors:** Sarah J. Firman, Jennifer Cook, Imogen Hall, Katie Yeung, Raphael Buttigieg, Fiona Vaz, Anthony S. Wierzbicki, Radha Ramachandran

**Affiliations:** ^1^ Department of Nutrition and Dietetics Guy's and St Thomas' NHS Foundation Trust London UK; ^2^ Adult Inherited Metabolic Diseases, Guy's and St Thomas' NHS Foundation Trust London UK

**Keywords:** adult Refsum disease, carbohydrate intake, dietary management, energy intake, phytanic acid

## Abstract

Adult Refsum disease (ARD, OMIM #266510) is an autosomal recessive condition, resulting in phytanic acid (PA) accumulation in plasma and tissue. The management of ARD relies on a low PA diet, but the importance of adequate energy and carbohydrate provision in reducing circulating PA levels and preventing metabolic crisis is less well described. Two patients recently diagnosed with ARD initiated on the low PA diet leading to a 65% reduction in circulating PA levels, one case achieving this reduction in 5 months. Four patients already established on the low PA diet for > 10 years experienced a rapid rise in circulating PA levels due to weight loss and/or inadequate carbohydrate intakes, despite adherence to a low PA diet. Circulating PA levels more than doubled in all four of these cases. Implementing dietary interventions to ensure adequate energy, carbohydrates and weight stabilisation resulted in a decrease in circulating PA levels by 47%–84% in the outpatient setting. The reduction in circulating PA occurred within 1.6–5 months. These cases demonstrate that PA restriction, and weight and carbohydrate management have integral roles in ensuring metabolic stability in ARD. This case series highlights that circulating PA levels can be reduced more rapidly with dietary interventions than previously shown in case series of chronic management.

**Summary**

- Adequate energy and carbohydrate intake are essential, alongside a low phytanic acid diet, in successfully reducing circulating phytanic acid levels and preventing metabolic crises in adult Refsum disease.

## 1 Introduction

Adult Refsum disease (ARD, OMIM #266510) is a rare, autosomal recessive condition caused by a defect in the breakdown of phytanic acid (PA), a branched‐chain fatty acid [1, 2]. The accumulation of PA in tissues and plasma results in a range of progressive neurological and systemic symptoms, including retinitis pigmentosa, anosmia, peripheral neuropathy, ataxia and hearing loss [2]. Given PA is obtained exclusively from food, dietary management is the mainstay of treatment [3].

Treatment aims to maintain low levels of plasma PA [3, 4]. This is achieved through following a low PA diet aiming to limit dietary PA intake to < 10 mg/day [3]. The low PA diet is devoid of dairy products, red meats and some seafoods. All patients in our centre are reviewed in clinic every 6–12 months, including monitoring of nutritional bloods and plasma PA levels. In cases with raised plasma PA levels (> 500 μmol/L) and/or acute symptoms of ARD, plasma PA levels are monitored weekly to monthly. Dietary sources of omega‐3 fatty acids (oily fish, fish oils, algae) need to be restricted due to their high PA content, potentially risking deficiencies in essential fatty acids (EFAs). Therefore, EFA are monitored and supplementation is provided as needed. No other nutritional supplements are routinely provided unless nutritional deficiencies are evident in biochemical monitoring or dietary assessments. All patients are provided with an oral emergency regimen containing glucose polymers to initiate during illness or periods of poor oral intake to prevent lipolysis and subsequent rising plasma PA levels.

A retrospective analysis of plasma PA levels in 13 patients with ARD who received dietary treatment over 10 years demonstrated the effectiveness of the low PA diet [5]. Plasma PA levels declined by a mean of 89% ± 11% (median 85, range 10–1325 μmol/L) after initiating the low PA diet. Blood PA levels were completely normalised (< 30 μmol/L) in 30%; partially normalised (30–300 μmol/L) in 50% and remained > 300 μmol/L in 15%. In addition to restricting PA, consuming adequate energy and carbohydrates is essential in optimising circulating PA levels [3, 4]. Periods of fasting, poor oral intake, or inadequate carbohydrates lead to lipolysis causing a release of PA from body tissue [3]. Fasting has been shown to result in a doubling in circulating PA levels within 29 h [6].

Wierzbicki et al. [6] reported one case where circulating PA levels halved in 22 days with acute in‐patient dietary management. In an outpatient chronic management setting, Baldwin et al. [5] demonstrated that the time required for circulating PA levels to halve was 44 ± 16 months in patients adherent to a low PA diet. Since this publication, the ARD dietary recommendations have been updated and are published on the Global DARE Foundation website [7]. We report two patient cases (Case Reports 1 and 2) who were newly diagnosed with ARD where initiating the updated dietary recommendations in an outpatient setting resulted in a rapid reduction in circulating PA levels. Furthermore, we report four patient cases (Case Reports 3–6) with known ARD already established on dietary treatment where weight loss, calorie deficits and inadequate carbohydrate intake led to a rapid rise in circulating PA levels despite compliance with the low PA diet. Circulating PA levels were successfully lowered by ensuring adequate energy and carbohydrate intake in addition to adherence to a low PA diet.

## 2 Outcome of Initiating Low Phytanic Acid Diet on Circulating Phytanic Acid Levels

The cases below demonstrate the impact of initiating a low PA diet at the diagnosis of ARD on circulating PA levels. This highlights the importance of dietary intervention to improve PA levels. Table 1 outlines patient characteristics, circulating PA levels at diagnosis and after initiating a low PA diet.

**TABLE 1:** Impact of starting a low phytanic acid diet on circulating phytanic acid levels in patients newly diagnosed with ARD.

Patient case	Sex	Age (years)	Age at diagnosis (years)	Age at commencement of diet (years)	Ethnicity	Body mass index (kg/m^2^)	Phytanic acid at diagnosis	Phytanic acid after diet initiation	Decrease (%)	Time period of decrease (months)
1	M	66	62	62	White—British	28.4	1067	377	65	27
2	F	61	59	60	Greek	24.7	719	254	65	5

### 2.1 Case Report 1

A 62‐year‐old male, newly diagnosed with ARD based on molecular genetics (compound heterozygous in the *PHYH* gene: c.135‐2A>G and c.526C>A, along with a variant of uncertain significance c.589G>C), initiated a low PA diet in accordance with level 1 dietary recommendations as per Global DARE Foundation [7]. This patient partook in regular exercise with 20–30 min on a static bike daily and 15 000 steps 4 days a week. Advice was also given regarding appropriate carbohydrate provision around exercise. Baseline circulating PA was 1067 μmol/L. After dietary interventions, circulating PA reduced to 624 μmol/L in 14 months and by 27 months, had reduced by 65% (Table 1).

### 2.2 Case Report 2

The second case is a 59‐year‐old female newly diagnosed with ARD after a serum PA level was taken following biochemically confirmed ARD in her siblings. Their presenting circulating PA was found to be 719 μmol/L (genetics pending). After initiating a low PA diet aligning with level 1 advice as per Global DARE Foundation dietary recommendations [7], PA levels reduced by 65% in 5 months (Table 1).

## 3 Impact of Weight Loss, Calorie Deficits and Inadequate Carbohydrate Intake on Phytanic Acid Control

The following four cases were diagnosed with ARD > 10 years ago and have been established on a long‐term low PA diet (Table 2). Despite adherence to low PA dietary advice, they all experienced a rapid rise in their circulating PA levels due to either calorie deficits resulting in weight loss or inadequate carbohydrate intake. This demonstrates the importance of preventing PA mobilisation from adipose tissue in addition to limiting exogenous sources of PA in the diet. All cases had a significant improvement in circulating PA levels and resolution of acute symptoms with dietary intervention in an outpatient setting. Table 2 outlines patient characteristics, baseline and post‐intervention PA levels and the time period for these reductions in levels.

**TABLE 2:** Impact of optimising carbohydrate and energy intake in patients already established on a low phytanic acid diet.

Patient case	Sex	Age (years)	Age at diagnosis (years)	Age at commencement of diet (years)	Ethnicity	Body mass index (kg/m^2^)	Acute phase circulating phytanic acid	Follow‐up phytanic acid	Decrease (%)	Time period of decrease (months)
3	M	72	30	30	White—British	25.5	557	91	84	1.6
4	F	39	10	10	British Indian	35.1	1078	442	59	4
5	F	49	37	37	Greek	21.5	1363	623	54	5
6	M	63	21	21	White—British	24.5	891	469	47	4

### 3.1 Case Report 3

A 72‐year‐old male with a biochemical diagnosis of ARD (sibling has ARD and has been genetically confirmed to have a homozygous splice mutation c.678+2T>G) developed worsening leg pain over 3 months and was subsequently diagnosed with polymyalgia rheumatica. Due to persistent pain, he experienced reduced appetite and intake resulting in a weight loss of 5 kg (8% of body weight) in 3 months. During this time, circulating PA increased from 200–300 to 557 μmol/L. Energy intake was optimised through oral nutritional supplements supporting gradual weight gain. With adequate energy intake, circulating PA levels reduced by 50% within 4 weeks and 84% within 7 weeks to 91 μmol/L (Table 2).

### 3.2 Case Report 4

A 39‐year‐old female with genetically confirmed ARD (homozygous for *PHYH* c.164delT) had self‐initiated diet and lifestyle changes to lose weight. The patient increased her physical activity to 45–60 min per day of aerobic exercise and consumed a low carbohydrate diet, leading to a 3.7 kg weight loss (4.4% weight loss) over 3 weeks. She reported acute symptoms of worsening vision, peripheral neuropathy and ataxia. Circulating PA levels had increased from 340 to 1078 μmol/L in 1 month. Implementing regular meals and increasing carbohydrate foods at all meals and snacks led to weight stabilisation and a reduction in circulating PA levels of 59% in 4 months (Table 2).

### 3.3 Case Report 5

A 49‐year‐old female with genetically confirmed ARD (Homozygous for *PHYH* c.790C>T) following a low PA diet reported acute symptoms of blurred vision and ataxia. Circulating PA was found to be 1363 μmol/L. On further investigation, this patient had been experiencing low appetite, reducing carbohydrate intake and weight loss of 4 kg (6.3%) in 5 months. Initiating regular meals (three main meals and three snacks), including a before‐bed snack, all containing adequate carbohydrates, resulted in a 1.5 kg weight gain over 4 months. Circulating PA reduced by 54% in 5 months with these dietary interventions (Table 2).

### 3.4 Case Report 6

A 63‐year‐old male with genetically confirmed ARD (Homozygous for *PHYH* c.678+2T>G) and reactive hypoglycaemia was informed by their general practitioner of a raised HbA_1c_ indicative of diabetes. The patient self‐initiated a lower carbohydrate diet in an effort to improve their HbA_1c_. Circulating PA increased from 124 to 891 μmol/L with the reduction in carbohydrate intake. Weight loss was 1.4 kg (2% of body weight) during this time. A continuous glucose monitor (CGM) was used to guide the reintroduction of carbohydrate food sources to ensure adequate carbohydrate provision for ARD while improving glucose control and preventing reactive hypoglycaemia. Low PA protein and fat food sources were added to meals to reduce the overall glycaemic index of meals. Dietary changes resulted in a reduction in circulating PA of 47% in 4 months (Table 2) and improvement in HbA_1c_ from 50 to 43 mmol/mol.

## 4 Discussion

This case series highlights the important role of adequate energy and carbohydrate provision, in addition to a low PA diet, in optimising circulating PA levels. Case reports 1 and 2 demonstrate that the initiation of a low PA diet on diagnosis of ARD, aligning with the Level 1 Global DARE Foundation dietary recommendations, can result in a reduction in circulating PA of > 50%, validating these dietary recommendations [7]. In addition to successfully reducing circulating PA levels, these dietary recommendations resulted in a more rapid reduction in circulating PA levels than has been previously described [5]. In a chronic management outpatient setting, Baldwin et al. [5] reported that the time required for circulating PA levels to halve was 44 ± 16 months in patients adhering to a low PA diet. For the presented case series, circulating PA levels reduced by > 50% in a time frame as short as 5 months. Those who described worsening acute symptoms with high plasma PA levels reported subjective improvements in symptoms with the rapid reduction in PA levels. Our centre aims to maintain lower PA levels to reduce metabolic risk in cases of intercurrent illness. Therefore, in cases where acute symptoms are not evident despite raised PA levels, dietary interventions would be initiated to bring PA levels down even when patients do not describe any worsening of symptoms.

Adherence to a low PA diet is essential in the management of ARD; however, weight and carbohydrate management also have integral roles in ensuring metabolic stability. As demonstrated in this case series, circulating PA levels can rapidly increase despite adherence to a low PA diet when there is inadequate energy and/or carbohydrate intake. Dietary interventions to ensure adequate energy for weight stabilisation and adequate carbohydrate provision can precipitate improvement in circulating PA levels. Patients were able to rapidly reduce circulating PA levels and prevent metabolic crisis without the need for inpatient management or plasmapheresis. However, this should be assessed on an individual basis with consideration for presenting symptoms and close monitoring.

The impact of weight loss on circulating PA levels has been demonstrated in this case series, which was further exacerbated by inadequate carbohydrate provision. Weight loss results from a state of calorie deficit, leading to lipolysis and mobilisation of PA from body tissues. Unintentional weight loss should be prevented with early intervention, including initiating an oral emergency regimen using a glucose polymer at the first sign of illness or poor oral intake, to prevent rising circulating PA levels [7]. Intentional weight loss may be indicated to support improved body composition and prevent co‐morbidities; however, weight loss should be very gradual whilst ensuring adequate carbohydrate provision and close monitoring of circulating PA levels. For patients with high circulating PA levels, weight loss is not recommended [7].

Case Report 6 highlighted that despite adherence to a low PA diet and with minimal weight loss, reducing carbohydrate intake resulted in a significant increase in circulating PA levels. As a result of this, managing diabetes with ARD can be challenging and needs close monitoring. To prevent rising PA levels, carbohydrate provision should meet the higher end of the acceptable macronutrient distribution range of 45%–65% of total energy intake [8] and feature as part of all meals and snacks. Therefore, the source of carbohydrates and incorporating low PA protein and fat food sources into meals is important to lower the glycaemic response. CGM is a beneficial tool for monitoring and improving diabetes control while implementing dietary changes to ensure adequate carbohydrate to optimise circulating PA levels.

## 5 Conclusion

This case series demonstrates the role of PA restriction in ARD, and that weight management and energy and carbohydrate provision have integral roles in ensuring metabolic stability. Implementing a low PA diet at the diagnosis of ARD can improve circulating PA levels, with levels halving in as little as 5 months. Close monitoring of weight, energy and carbohydrate intake is essential, as deficits in these can lead to a rapid rise in circulating PA levels and risk of metabolic crisis. Furthermore, early initiation of an emergency regimen using glucose polymer during illness is essential to prevent the risk of metabolic crisis.

## Author Contributions

Sarah J. Firman and Radha Ramachandran were involved in the conceptualisation of this case series. Sarah J. Firman led on data collection and analysis and interpretation, along with Radha Ramachandran. The original draft was prepared by Sarah J. Firman. Radha Ramachandran, Anthony S. Wierzbicki, Jennifer Cook, Imogen Hall and Katie Yeung provided critical revision of the draft. All authors revised and approved the final version of the manuscript submitted for publication.

## Ethics Statement

No ethical approval was required to undertake this retrospective case review.

## Consent

The authors have obtained written informed consent using the Wiley's consent form from all patients included in this publication.

## Conflicts of Interest

Sarah J. Firman, Radha Ramachandran and Anthony S. Wierzbicki are all members of the Medical & Scientific Advisory Board for the Global DARE Foundation. The other authors declare no conflicts of interest.

## Data Availability

The data that support the findings of this study are available from the corresponding author upon reasonable request.

## References

[1] R. J. Wanders , J. Komen , and S. Ferdinandusse , “Phytanic Acid Metabolism in Health and Disease,” Biochimica et Biophysica Acta 1811, no. 9 (2011): 498–507, 10.1016/j.bbalip.2011.06.006. 21683154

[2] A. S. Wierzbicki , M. D. Lloyd , C. J. Schofield , M. D. Feher , and F. B. Gibberd , “Refsum's Disease: A Peroxisomal Disorder Affecting Phytanic Acid Alpha‐Oxidation,” Journal of Neurochemistry 80, no. 5 (2002): 727–735, 10.1046/j.0022-3042.2002.00766.x. 11948235

[3] E. Baldwin , “Refsum's Disease,” in Manual of Dietetic Practice, 5th ed., ed. J. Gandy (British Dietetic Association, 2014), 589–593.

[4] H. Waterham , R. J. Wanders , and B. P. Leroy , “Adult Refsum Disease,” in Gene Reviews, ed. Adam MP , Feldman J , Mirzaa GM , et al., (University of Washington, 2006), 1993–2025, https://www.ncbi.nlm.nih.gov/books/NBK1353/. 20301527

[5] E. J. Baldwin , F. B. Gibberd , C. Harley , M. C. Sidey , M. D. Feher , and A. S. Wierzbicki , “The Effectiveness of Long‐Term Dietary Therapy in the Treatment of Adult Refsum Disease,” Journal of Neurology, Neurosurgery & Psychiatry 81, no. 9 (2010): 954–957, 10.1136/jnnp.2008.161059. 20547622

[6] A. S. Wierzbicki , P. D. Mayne , M. D. Lloyd , et al., “Metabolism of PA and 3‐Methyl‐Adipic Acid Excretion in Patients With Adult Refsum Disease,” Journal of Lipid Research 44, no. 8 (2003): 1481–1488, 10.1194/jlr.M300121-JLR200. 12700346

[7] DARE , “Refsum Diet: Global Dare Foundation,” 2024, https://www.defeatadultrefsumeverywhere.org/refsum‐diet.

[8] IOM , Dietary Reference Intakes for Energy, Carbohydrate, Fiber, Fat, Fatty Acids, Cholesterol, Protein, and Amino Acids (National Academies Press, 2005). 10.1016/s0002-8223(02)90346-9 12449285

